# Perspectives on Medical Education Meta-Research Special Issue: A call for papers exploring how research is performed, communicated, verified and rewarded

**DOI:** 10.1007/s40037-020-00627-8

**Published:** 2020-11-09

**Authors:** Lauren A. Maggio, Stefanie Haustein, Anthony R. Artino Jr.

**Affiliations:** 1 Uniformed Services University of the Health Sciences, Bethesda, MD USA; 2 University of Ottawa, Ottawa, ON Canada; 3 The George Washington University School of Medicine and Health Sciences, Washington, DC USA

A recent PubMed search for “medical education” retrieved 192,174 articles with over 7500 published in 2020 thus far. In this same period, the journal *Perspectives on Medical Education* has received over 500 manuscripts for peer review. At a glance, these numbers suggest medical education is a burgeoning field, and from our perspective it certainly is. However, alone these growing numbers provide limited information about *how* medical educators perform, communicate, and verify the field’s research or how the field rewards research. When we talk about understanding these topics, we are squarely in the realm of meta-research or the study of research itself [1, 2].

In science broadly, meta-research (aka science of science and related fields like bibliometrics and scientometrics) has been proposed as critical to increasing the effectiveness and value of scientific research, as well as improving its conduct and the research culture surrounding it [1, 3]. At *Perspectives on Medical Education*, we believe the time is now for medical education to conduct meta-research on our field to critically examine our own research and research practices. Thus, the Journal is launching a special meta-research issue and releasing this call for manuscripts.

We recognize that meta-research is a broad domain. Thus, Tab. 1 provides some sample topics. References [1] and [3] provide insights into meta-research and cite papers that might be helpful models.

**Table 1:** Sample topics to be explored around how research is performed, communicated, verified and rewarded in medical education

Performed	Open science practices (open access, data sharing, open peer review, study registration)
	Reporting guidelines
	Responsible research conduct
Communicated	Preprints
	Open access
	Academic writing and language use
	Social media in scholarly communication
Verified	Peer review
	Reproducibility
	Predatory publication
Rewarded	Promotion and tenure
	Scholarly metrics including bibliometrics, altmetrics, patent metrics, data metrics

For the special issue, we welcome manuscripts that describe the conduct of meta-research in medical education using quantitative, qualitative, or mixed-methods approaches. Knowledge syntheses on meta-research topics and bibliometric analyses are also welcome. In addition, to facilitate the future use and conduct of research, we are interested in descriptions of courses and educational activities that have been implemented and evaluated to promote meta-research in medical education and related open educational resources (OERs).

## Format

Manuscripts should not exceed 3500 words and generally include up to five black and white and/or color exhibits (e.g., figures, tables). However, we recognize that meta-research manuscripts tend to have robust and at times lengthy methods sections. Therefore, in addition to the 3500-word manuscript, we invite authors to also submit a detailed methods section (if appropriate), which we will collate into a methods collection hosted on Zenodo, an open access repository for research-related digital objects. We hope that the methods collection can serve as a stand-alone OER and develop into a “cookbook” for researchers interested in applying meta-research methodologies to explore and analyze research outputs and scholarly communication. Please follow the journal’s guidelines for original research. Abstracts are limited to 250 words. For full author instructions, see: https://www.springer.com/journal/40037/submission-guidelines.

## Submission, review and publication process

Manuscripts will be accepted on a rolling basis, but for full consideration, authors must submit their manuscripts by June 30, 2021. All manuscripts must be submitted using the journal’s editorial manager system.

Upon submission, authors should select “Special Issue: Meta-research” as the publication type and note in their cover letter that the submission is in response to this call for papers. Per the journal’s policy, we *encourage* authors to deposit a preprint of their work prior to submission [4].

All manuscripts will first be screened by the guest editors (Drs. Lauren Maggio, Stefanie Haustein, and Anthony Artino). Manuscripts passing the screening phase will then be sent for external peer review.

## Publication process

As with all *Perspectives on Medical Education* articles, once a manuscript is accepted for publication it will be published gold open access (i.e., freely accessible, without an author processing fee) e‑pub ahead of print to the journal’s website and its metadata transmitted to multiple databases (e.g., PubMed, PMC, Scopus, etc.). In the Winter of 2021–2022, the guest editors will collate all articles into a special issue of the journal.
