# Correction to: perspectives on medical education meta-research special issue: a call for papers exploring how research is performed, communicated, verified and rewarded

**DOI:** 10.1007/s40037-020-00640-x

**Published:** 2020-12-14

**Authors:** Lauren A. Maggio, Stefanie Haustein, Anthony R. Artino

**Affiliations:** 1 Uniformed Services University of the Health Sciences, Bethesda, MD USA; 2 University of Ottawa, Ottawa, ON Canada; 3 School of Medicine and Health Sciences, The George Washington University, Washington, DC, USA

**Correction to:**

**Perspect Med Educ 2020**

10.1007/s40037-020-00627-8

The original version of this article unfortunately contained a mistake. References [1, 2] and [4] were incorrect. The corrected references are given below. The original article has been corrected.
